# A Four-Point Screening Method for Assessing Molecular Mechanism of Action (MMOA) Identifies Tideglusib as a Time-Dependent Inhibitor of *Trypanosoma brucei* GSK3β

**DOI:** 10.1371/journal.pntd.0004506

**Published:** 2016-03-04

**Authors:** Zachary T. Swinney, Brad A. Haubrich, Shuangluo Xia, Chakk Ramesha, Stephen R. Gomez, Paul Guyett, Kojo Mensa-Wilmot, David C. Swinney

**Affiliations:** 1 Institute for Rare and Neglected Diseases Drug Discovery, Mountain View, California, United States of America; 2 Department of Cellular Biology and Center for Tropical and Emerging Global Diseases, University of Georgia, Athens, Georgia, United States of America; Institute of Tropical Medicine, BELGIUM

## Abstract

**Background:**

New therapeutics are needed for neglected tropical diseases including Human African trypanosomiasis (HAT), a progressive and fatal disease caused by the protozoan parasites *Trypanosoma brucei gambiense* and *T*. *b*. *rhodesiense*. There is a need for simple, efficient, cost effective methods to identify new molecules with unique molecular mechanisms of action (MMOAs). The mechanistic features of a binding mode, such as competition with endogenous substrates and time-dependence can affect the observed inhibitory IC_50_, and differentiate molecules and their therapeutic usefulness. Simple screening methods to determine time-dependence and competition can be used to differentiate compounds with different MMOAs in order to identify new therapeutic opportunities.

**Methodology/Principal Findings:**

In this work we report a four point screening methodology to evaluate the time-dependence and competition for inhibition of GSK3β protein kinase isolated from *T*. *brucei*. Using this method, we identified tideglusib as a time-dependent inhibitor whose mechanism of action is time-dependent, ATP competitive upon initial binding, which transitions to ATP non-competitive with time. The enzyme activity was not recovered following 100-fold dilution of the buffer consistent with an irreversible mechanism of action. This is in contrast to the *T*. *brucei* GSK3β inhibitor GW8510, whose inhibition was competitive with ATP, not time-dependent at all measured time points and reversible in dilution experiments. The activity of tideglusib against *T*. *brucei* parasites was confirmed by inhibition of parasite proliferation (GI_50_ of 2.3 μM).

**Conclusions/Significance:**

Altogether this work demonstrates a straightforward method for determining molecular mechanisms of action and its application for mechanistic differentiation of two potent TbGSK3β inhibitors. The four point MMOA method identified tideglusib as a mechanistically differentiated TbGSK3β inhibitor. Tideglusib was shown to inhibit parasite growth in this work, and has been reported to be well tolerated in one year of dosing in human clinical studies. Consequently, further supportive studies on the potential therapeutic usefulness of tideglusib for HAT are justified.

## Author Summary

Drug discovery for neglected tropical diseases must use efficient methods due to limited resources. One preferred drug discovery strategy is target-based drug discovery. In this strategy it is assumed that drug action begins with binding of a drug to its target. However, while binding is required, it is not sufficient to describe all the molecular interactions that translate binding to a therapeutically useful response. The contribution of aspects of the molecular mechanism of action (MMOA) such as time-dependence and substrate competition can influence concentration response relationships. To address this, a four point MMOA methodology was developed to evaluate time-dependence and substrate competition. We used this method to evaluate the MMOA for *T*.*brucei* GSK3β inhibitors, and observed tideglusib to have a time-dependent, ATP-competitive mechanism that differentiated it from rapidly reversible inhibitors, such as GW8510. Adjusting the enzyme assays to account for these mechanisms showed that GW8510 and tideglusib had similar activities for TbGSK3β. However, this similarity did not translate to cellular activity, where GW-8510 was more active than tideglusib (0.12 μM to 2.3 μM, respectively). These data suggest that factors other than TbGSK3β MMOA differentiate the effect of these molecules against *T*. *brucei*.

## Introduction

New medicines are needed for neglected tropical diseases (NTDs). Drug discovery and development in all disease areas is inefficient due to high failure rates that increase the costs. The impact of the high failure rates on NTDs is very significant as a result of limited resources afforded to these diseases because of a lack of commercial markets for the medicines [1]. There is an urgent need for cost effective methods and strategies that expedite opportunities to identify new medicines for NTDs [1].

As part of our on-going efforts to identify new treatment molecules for human African trypanosomiasis (HAT; also known as sleeping sickness) we have been investigating approaches to identify new molecular mechanisms of action (MMOAs) early in the discovery process. HAT is a deadly infection affecting mostly impoverished areas in rural sub-Saharan Africa, caused by the protozoan *Trypanosoma brucei*. There are currently an estimated 30000 cases of HAT, with approximately 70 million people in 36 African countries at risk [2]. At this time, five medicines are available for HAT, all of which must be administered intravenously or intramuscularly, and three of these treat late-stage infections, when the parasite has crossed the blood-brain barrier. Notable limitations of these treatments include ineffectiveness, long periods of drug administration, cost, toxicity, and other severe medical side effects [3]. Consequently, there is a critical need to discover and develop safer, more effective and less expensive therapies for HAT. There is hope that this unmet medical need will be addressed with the promising candidates currently in development, fexinidazole and SCYX-7158 [4]. However, there is still a need for back-up strategies for these compounds in case they do not meet the promise and to also address potential resistance.

Drug discovery and development is facilitated by an understanding of how drugs work (i.e. their mechanism of action). How a drug works is a key feature of the pharmacological response, the therapeutic index, and the drug’s therapeutic usefulness [5–7]. Knowledge of a drug’s mechanism will also facilitate optimization and clinical development. It has been long recognized that pharmacological action begins with an interaction between two molecules. Ehlrich noted in 1913 that a substance will not work unless it is bound “*corpora non agunt nisi fixata*” [8]. However, binding alone is not sufficient for a medicine to produce an effective and safe response that is therapeutically useful. The molecular mechanism of action (MMOA) through which a medicine connects binding to the response is also important [9, 10]. For example, two similarly structured molecules can bind to an enzyme with similar affinity; however, one will bind as a substrate in a manner suitable for the catalytic reaction while the other will inhibit the reaction. For receptors, two similarly structured molecules can bind to a receptor with similar affinity; however, one will initiate the response (agonist) whereas the other will block the response (antagonist).

Understanding drug action at the molecular level can facilitate the rational design of new medicines as well as provide opportunities to identify new therapies. Understanding the MMOA can provide opportunities to identify new molecules that are differentiated from previous molecules, molecules that can be repurposed for new indications or molecules that have been previously overlooked. A detailed understanding of the MMOA is also important for interpretation of *in vitro/ in vivo* correlations in target validation studies and understanding pharmacokinetic/pharmacodynamics (PK/PD) relationships.

Two important features of MMOA that have been shown to differentiate medicines are binding kinetics and binding competition. The binding kinetics are the rate at which a molecule binds (association rate) and debinds (dissociation rate). A reaction with a slow dissociation rate can be functionally irreversible when the dissociation rate is sufficiently slow or covalent. Competition occurs when two molecules compete for the same binding site and will result in decreased fractional occupancy of the drug bound to the target. The decrease in fractional occupancy due to competition can be overcome by increasing the concentration of the drug. The decrease in fractional occupancy due to competition can also be overcome with slow dissociation kinetics and irreversibility. This pharmacological behavior is described as insurmountable drug action.

Many examples demonstrate the important role of binding kinetics in effective drug action [9, 11, 12]. Aspirin is an irreversible inhibitor of prostaglandin H_2_ synthases (also known as cyclooxygenase, COX), whereas ibuprofen is a rapidly reversible inhibitor of these enzymes with a fast dissociation rate [13, 14]. The irreversibility of aspirin contributes to its usefulness for prevention of atherothrombotic disease [15, 16] and differentiates aspirin from ibuprofen. Irreversibility can be achieved by covalent binding as well as long residence times in a system not at equilibrium to provide insurmountable pharmacological behavior [17]. Slow dissociation kinetics in a system not at equilibrium contributes to the use-dependence behavior of channel blockers [18] and the insurmountable behavior of many receptor blockers, including the well-documented, angiotensin receptor blockers [19, 20]. These examples illustrate some of the advantages to time-dependent behavior including a greater inhibition of activity and longer lasting pharmacodynamic behavior and target occupancy enabling administration of lower doses and in some cases greater durability. These mechanistic behaviors contribute to the effectiveness and utility of many anti-infectives including the irreversible inhibitor, penicillin [21], and isoniazid [22, 23]. This behavior also contributes to the effectiveness of many other medicines including lapatinib, tiotropium, and candesartan to name a few [9, 11]. For completeness it must be noted that long-residence time/irreversibility is not suited for all system. When there are liabilities due to mechanism-based toxicity (on-target toxicity), long residence time/irreversible behavior is not appropriate [5, 9].

Competition of a drug with endogenous substrate for binding will reduce the fractional occupancy and may result in a loss of effectiveness. This will require higher concentrations to achieve the same effect and thereby decrease the selectivity, increase the potential for toxicity as well as provide a challenge for pharmaceutical development to administer the drug at a sufficient dose and concentration at the site of action to achieve efficacy.

Competition with endogenous substrates is particularly relevant for protein kinases where ATP competitive inhibitors must compete with high concentrations of endogenous ATP for binding and inhibition of the kinase activity. The physiological concentrations of ATP are estimated to be in excess of 1 mM, which will result in a ≈ 100 fold shift in IC_50_ for inhibitors with a Km for ATP of 10 μM (see theoretical explanation below). Importantly, mechanisms that avoid competition with ATP have been identified, including slow dissociation kinetics (long residence times) and non-competitive mechanisms. Wilson and coworkers recently demonstrated that the MMOA of the first approved kinase inhibitor, Gleevec (imatinib mesylate), involves time-dependent binding that is important to its action and selectivity [24]. This time-dependent, mechanistic behavior was not identified until over a decade after the drug had been discovered. In general, the many molecular mechanistic features important to the action of first in class drugs are identified long after a drug is discovered, and consequently, not used to inform optimization and development [6]. Accordingly, a simple, cost-effective method to identify MMOAs for lead compounds will be valuable to R&D, notably in resource limited diseases, such as NTDs.

**Fig 1:**
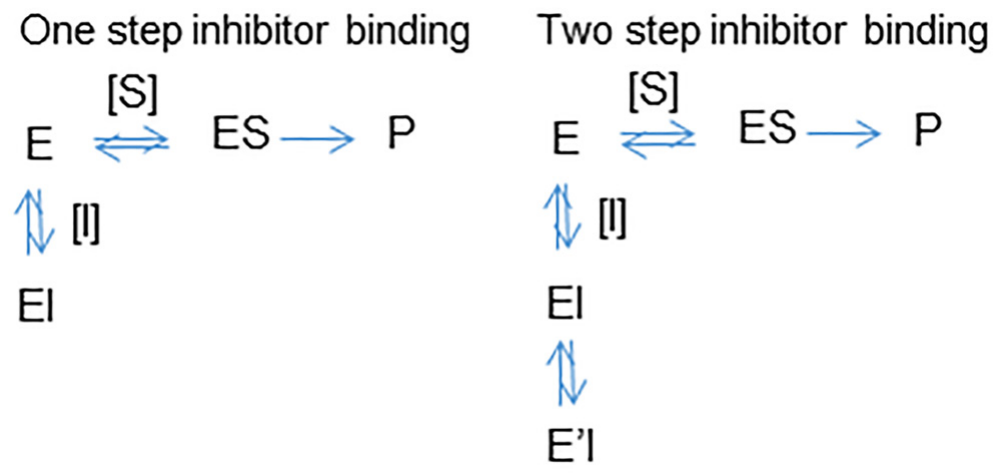
Kinetic scheme for slow binding kinetics. One-step binding: the inhibitor [I] and substrate [S] are in equilibrium; binding is competitive and mutually exclusive. Increasing [S] will shift the equilibrium to product formation and reduce the amount of enzyme in bound to inhibitor (EI); for two-step binding the formation of the E’I complex is time dependent and not in rapid equilibrium with ES; increasing [S] will have a smaller effect on reducing the fraction of enzyme in the E’I complex. This behavior looks noncompetitive and is termed insurmountable.

Towards this goal, we have used a simple four point method to characterize the MMOA of compounds for time-dependence and competition as part of our efforts to identify novel and therapeutically useful protein kinase inhibitors of *T*. *brucei*. The method involves measuring activity at one inhibitor concentration with and without a preincubation (30 min in this case) to determine time-dependence, and at two substrate concentrations (0.5 Km and 5x Km in this work) to determine competition. A shift in the time dependence indicates that the inhibitor binding does not reach equilibrium in the time frame of the experiment. The inability to rapidly reach equilibrium can be due to a multi-step binding mechanism (Fig 1). A loss of activity with higher substrate concentrations is consistent with a substrate competitive mechanism of inhibition. A lack of shift in inhibition can be considered as noncompetitive, such as inhibitor binding in a different site. However, a combination of noncompetitive behavior and time-dependence can be diagnostic of a molecule with insurmountable pharmacological behavior due to slow binding kinetics (including irreversibility) that prevents competition. For example, an irreversible inhibitor will appear noncompetitive even when it is bound in the substrate binding site.

The utility of the four point MMOA method was demonstrated with GSK3β isolated from *T*. *brucei* (TbGSK3β) and the identification of tideglusib (NP-12, NP031112) as a time-dependent, competitive inhibitor of TbGSK3β. Further evaluation of tideglusib showed irreversible behavior against the enzyme and inhibition of parasite proliferation at low micromolar concentrations. Tideglusib, previously evaluated in the clinic, has a good safety profile in humans [25, 26] and therefore, warrants further studies to evaluate its potential for HAT. Altogether this work demonstrates a method for rapidly evaluating MMOA that can help identify opportunities for new NTDs.

## Materials and Methods

### Materials

Reagents, unless noted, were purchased from Sigma-Aldrich (Saint Louis, Missouri). GSM peptide (sequence = RRRPASVPPSPSLS RHS(pS)HQRR, where pS is a phosphorylated serine residue) [27] was purchased from EMD Millipore Corporation (Temecula, California). ADP-Glo kit was purchased from Promega Corporation (Madison, Wisconsin). Tideglusib and GW8510 were purchased from Sigma-Aldrich (Saint Louis, Missouri).

### Methods

#### Expression and purification of recombinant TbGSK3β

Full-length TbGSK3β (Gene DB ID Tb927.10.13780; UniProt Accession number UniProtKB—Q388M1) was cloned into the pET21d expression vector in-frame with a C-terminal 6X His tag. BL21 (DE3) *E*. *coli* were transformed with the construct and cultured in terrific broth (TB) supplemented with 1% glucose to an OD (at 590 nm) of 1.8. Expression of TbGSK3β was induced with IPTG (0.5 mM) for 3 hours at 32°C. Cells were harvested, and resuspended in lysis buffer (30 mM HEPES (pH 8.0), 300 mM NaCl, 10% glycerol, 10 mM imidazole, 0.2 μM PMSF, and 2 mM β-mercaptoethanol). The bacteria were lysed by sonication and freeze/thaw cycles. Insoluble protein were removed by centrifugation (12000 x g, 45 min, 4°C), and the soluble fraction loaded onto immobilized metal affinity chromatography (IMAC) column. The column was washed with 30 mM HEPES (pH 8.0), 0.5 M NaCl, 10% glycerol, 20 mM imidazole, 0.02% Triton X-100, 2 mM β-mercaptoethanol. Recombinant TbGSK3β (TbGSK3β) was eluted from the IMAC column with 6 mL of 30 mM HEPES (pH 7.5), 300 mM NaCl, 10% glycerol, 200 mM imidazole, 2 mM β-mercaptoethanol. Eluted TbGSK3β was further fractionated by size-exclusion chromatography (SEC) using a Superdex-200, 10/300 GL column pre-equilibrated with SEC running buffer, 50 mM Tris-HCl (pH 7.5), 100 mM NaCl. Fractions were collected, a portion of each was analyzed by SDS-PAGE to determine which fraction contained TbGSK3β. A band corresponding to the molecular weight of rTbGSK3β eluted in fractions 13–15, and was considered the purified enzyme. From a 12 x 1 liter of culture 136 mg of protein was recovered at 3.8 mg/ml. Purified TbGSK3β was stored at -80°C in 50 mM Tris-HCl (pH 7.5), 100 mM NaCl, 20% glycerol until used.

#### Enzyme assay

TbGSK3β activity was measured by following the ADP formation associated with the phosphorylation of GSM peptide and detected with ADP-Glo at 23°C. The enzyme reaction was conducted in a volume of 20 μL in a buffer containing 20 mM NaCl, 15 mM HEPES pH 7.4, 1 mM EGTA, 10 mM MgCl_2_, 0.02% Tween. These assays contained GSM at 10*μ*M (approximating Km) or 80 μM (approximating Vmax). ATP was added at 10 μM and 100 *μ*M to approximate Km and Vmax, respectively.

All reactions were incubated for 5 minutes in microcentrifuge tubes, and reactions were terminated at 80°C for 4 min. Of 20 μL reactions, 17.5 μL were transferred into 384 well-plates. Promega ADP-Glo procedures were carried out and phosphorylation measured by Luminoskan Ascent (Thermo Labsystems, Waltham MA). For pre-incubation experiments, TbGSK3β was preincubated at room temperature with inhibitor, GSM and buffer for 30 min. Inhibitors were added in DMSO to a final concentration of DMSO in the reactions of <5%. The kinase reaction was then started by adding ATP. For non-preincubation reactions, the kinase reactions were started by adding TbGSK3β to a mixture containing inhibitor, ATP, GSM and buffer. DMSO was added to all control reactions. All compounds evaluated in this study were tested for their ability to inhibit the ADP-Glo detection assay in experiments with ADP present and no TbGSK3β and did not inhibit this reaction up to 100 μM.

#### Parasite assay

Bloodstream form *Trypanosoma brucei* (3 x 10^3^ trypanosomes/mL) (subspecies *Trypanosoma brucei brucei*, strain CA427) were cultured in 24 well plates. Inhibitor was dissolved in DMSO to various stock concentrations then added to the trypanosome cultures (final DMSO concentration was 0.1%). Trypanosomes were exposed to inhibitor for 48 hours, and then counted using a Coulter Counter (100 μm aperture tube, monitoring particles between 2.5 and 5 μm). The dose-response on trypanosome growth was analyzed using a non-linear regression fit in GraphPad Prism 6. The tideglusib GI_50_ assay was conducted over 48 hours. In that time, control trypanosomes (DMSO-treated) proliferated from 4x10^3^ cells/mL to 7.3x10^5^ cells/mL.

### Theoretical

#### Time-dependent binding

Binding is initiated when two molecules form a complex, such as a small molecule (substrate or inhibitor) binding with an enzyme (E+ I → EI) (Fig 1). Binding kinetics are the rate of drug association and dissociation described by the rate constants, k_on_ and k_off_, respectively. The association rate is concentration dependent, while the dissociation rate is concentration independent. The ratio of dissociation rate constant to association rate constant is equal to the drug's equilibrium dissociation constant (K_I_ = k_off_/k_on_) and is used to determine the fraction of drug bound at specific total drug concentrations (defined as [drug]/([drug] + K_I_) and also known as the fractional occupancy). When inhibition of product formation is rapid and observed immediately following simultaneous addition of inhibitor and substrate, the inhibitor and substrate binding are in equilibrium. The contribution of a longer lived and more stable complex, (E’I), formed by conformational rearrangement of the initial complex (EI) can be detected experimentally following a preincubation of the enzyme and inhibitor prior to addition of substrate (Fig 1). The preincubation provides the time for EI to transition to E’I. For simplicity, we will assume that the inhibition associated with no preincubation is equivalent to EI and inhibition associated with preincubation is associated with E’I. The formation of the E’I complex can be reversible or irreversible.

#### Competition

Competition occurs when the binding of two molecules (substrate and inhibitor) to the same protein is mutually exclusive (when one is bound the other is not). Competition of an inhibitor with substrate under equilibrium conditions will decrease the amount of inhibitor bound (fractional occupancy) and decrease the amount of inhibition. The higher the substrate concentration, the less inhibition. The loss of inhibitory activity at equilibrium will depend on the relative concentrations of the inhibitor and substrate as well as their respective equilibrium constants. The effect of a competitive inhibitor on the rate of an enzyme reaction is described by the following relationship.

$${\text{v}}_{\text{o}}=\text{ Vmax}*\left[\text{S}\right]\text{/}\left(\left[\text{S}\right]\;+\text{ Km}\left(\text{1+}\left[\text{I}\right]{\text{/K}}_{\text{I}}\right)\right)$$ (1)

where v_o_ is the enzyme rate (velocity), Vmax is the maximal velocity, [S] is the substrate concentration, Km is the Michaelis constant for the substrate, [I] is the inhibitor concentration and K_I_ is the equilibrium dissociation constant of the inhibitor. The reaction velocity for a competitive inhibitor will change at different substrate concentrations and will depend on the ratios of [I]/K_I_ and [S]/Km. This change can be theoretically calculated by the following equation derived from the equation for competitive inhibition,

$${\text{v}}^{\text{1}}{\text{/v}}^{\text{2}}=\;\left({\text{S}}^{\text{1}}+\text{ Km}\left({1+\text{ I}/\text{K}}_{\text{I}}\right)\right)/\;\left({\text{S}}^{\text{2}}+\text{ Km}\left({1+\text{ I}/\text{K}}_{\text{I}}\right)\right)$$ (2)

where S^1^ and S^2^ are the two substrate concentrations; v^1^ and v^2^ are the respective velocity of product formation. For the studies in this report we run the kinase assays with substrates at approximately 0.5 Km (10 μM for ATP) and 5x Km (100 μM for ATP). We chose 100 μM ATP because the ADP-Glo assay does not work well above 100 μM ATP and 10 μM ATP then gives us a better window. At inhibitor concentrations near K_I_ (approximately the IC_50_ at low substrate concentration) then I/K_I_ = 1 and (1 + I/K_I_) = 2. The theoretical shift for a competitive inhibitor in this system where the Km is 20 μM and ATP concentrations are 10 and 100 μM is 3.7. When there is no competition, no reduction of inhibition will be observed between the two ATP concentrations (non-competitive behavior).

## Results

### Activity of TbGSK3β

**Fig 2:**
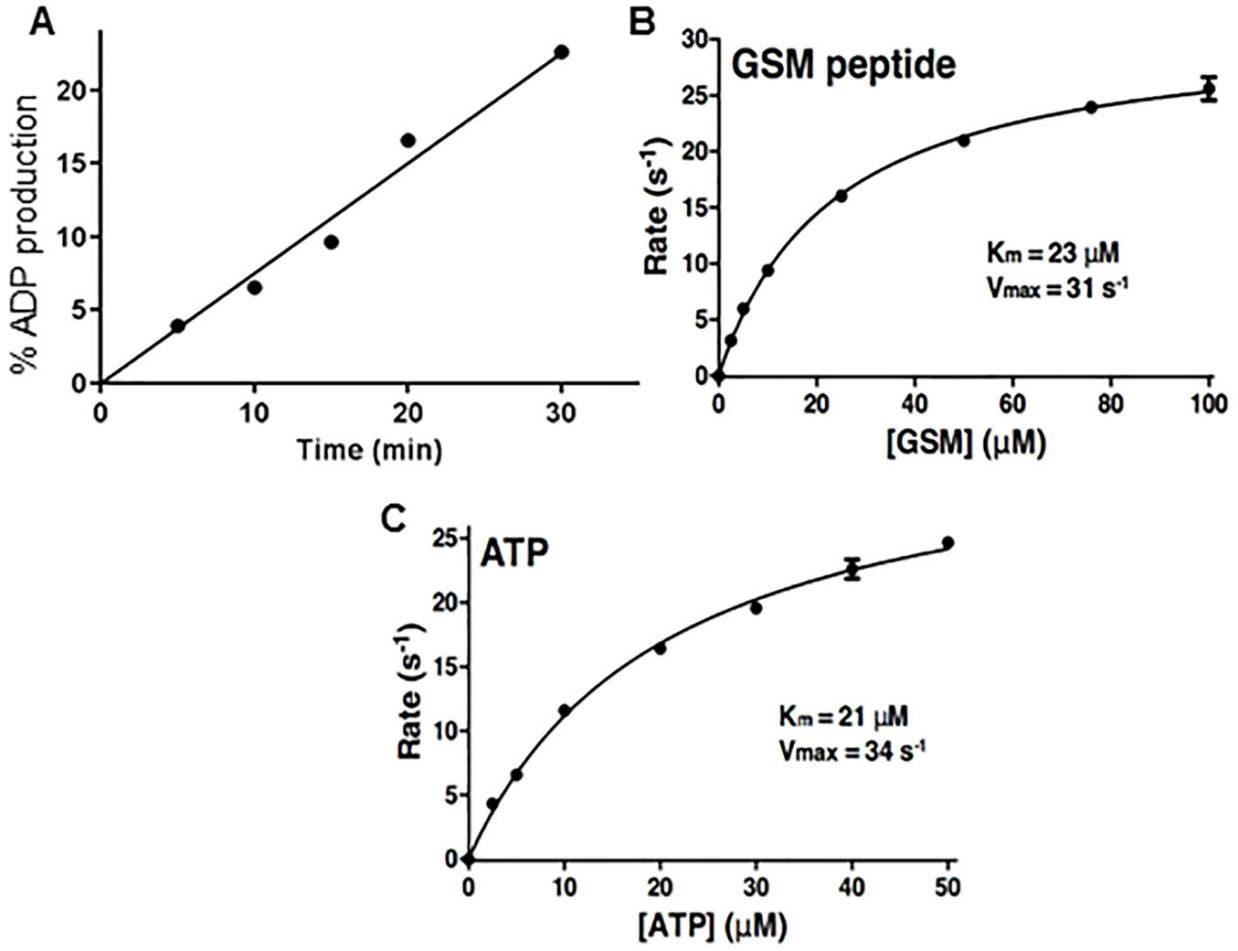
Substrate kinetics for TBGSK3β. Panel A. Time course of TbGSK3β, 1.5 nM, run in kinase buffer (15 mM HEPES, pH 7.4, 10 mM MgCl_2_, 1 mM EGTA, 0.02% Tween 20) with 10 μM ATP and 10 μM GSM. Reactions were initiated with the addition of enzyme to a total volume of 20 μL. Microtubes were mixed and sealed, and reactions were terminated by placement of the tube in a heat block for 5 min. Reaction mixtures were transferred to a 384-well plate, and ADP-Glo (Promega) detection was used according to the manufacturer’s instructions. Panels B and C. Michaelis Menten plots of reaction velocity with increasing concentrations of (B) GSM peptide and (C) ATP. They velocities are calculated from the nmol of ADP formed/nmol of enzyme (TbGSK3β)/ time.

The short-form of GSK3β from *T*. *brucei* was expressed in *E*. *coli* with a C-terminal His tag and purified to homogeneity as described in Materials and Methods. Conditions for the enzyme assay were optimized for linearity with time and enzyme concentration using GSM as a phosphoryl acceptor and measuring ADP production using Promega ADP-Glo reagents. The substrate versus rate plots showed saturable kinetics for the two substrates GSM and ATP, with Kms of 23 μM and 21 μM, respectively and Vmax of 31 and 34 s^-1^ in the two studies (Fig 2).

### Inhibition of TbGSK3β by tideglusib and GW8510

**Fig 3:**
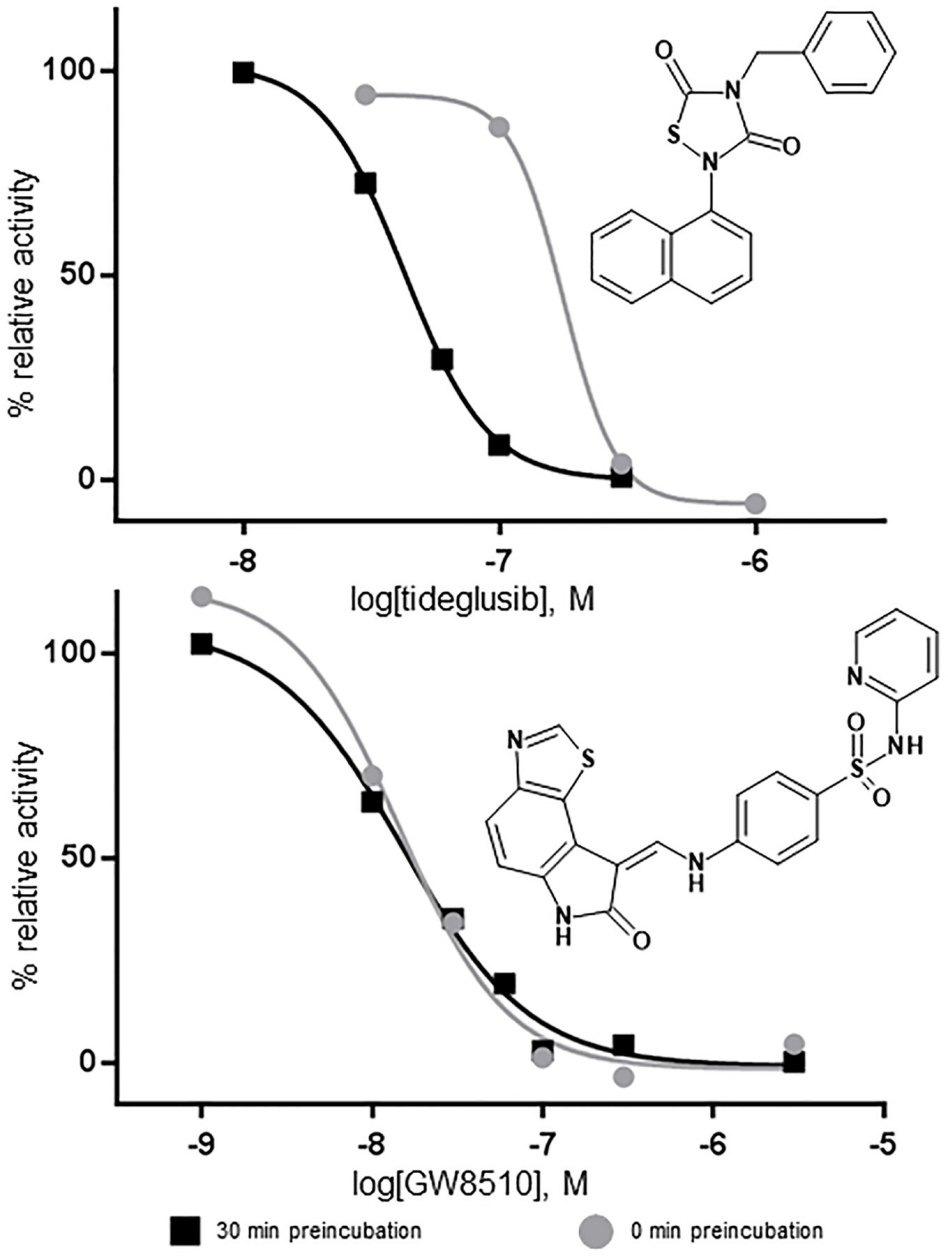
IC_50_ shift of inhibitors of TbGSK3β as a function of preincubation time with drug and kinase (10 nM) at 10 μM GSM peptide substrate and 10 μM ATP. Preincubated reactions were preincubated with inhibitor, TbGSK3β, GSM peptide, and buffer at room temperature; after 30 minutes reactions were initiated with the addition of ATP. Reactions with 0 min preincubation were initiated with the addition of TbGSK3β. Reactions were run for 5 min at room temperature, and stopped heat at 80°C, followed by ice bath, and ADP product formation was measured by ADP-Glo kit. All concentrations of tideglusib and controls with and without TbGSK3β were kept at 1% DMSO. Black = 30 minute preincubation Grey = 0 minute preincubation. (A) tideglusib IC_50_s 180 nM and 43 nM (B) GW8510, IC_50_s 14.5 nM and 15.1 nM

IC_50_ for inhibition TbGSK3β was determined as a function of preincubation time (Fig 3). Preincubated reactions were preincubated with inhibitor, TbGSK3β, GSM peptide, and buffer at room temperature; after 30 minutes reactions were initiated with the addition of ATP. Reactions with 0 min preincubation were initiated with the addition of TbGSK3β. Reactions were run for 5 min at room temperature, and stopped at 80°C. ADP product formation was measured by ADP-Glo kit. As shown in Fig 3 a shift in IC_50_ with preincubation time was observed for tideglusib, but not for GW8510. The IC_50_ values for tideglusib were 43 nM and 173 nM for preincubated reactions and non-preincubated reactions, with standard errors of 6.5 nM (N = 2) and 22.6 nM (N = 5), respectively. The IC_50_ values for GW-8510 were 14.5 nM and 15.1 nM for preincubated reactions and non-preincubated reactions, respectively.

### Four point MMOA assay

**Fig 4:**
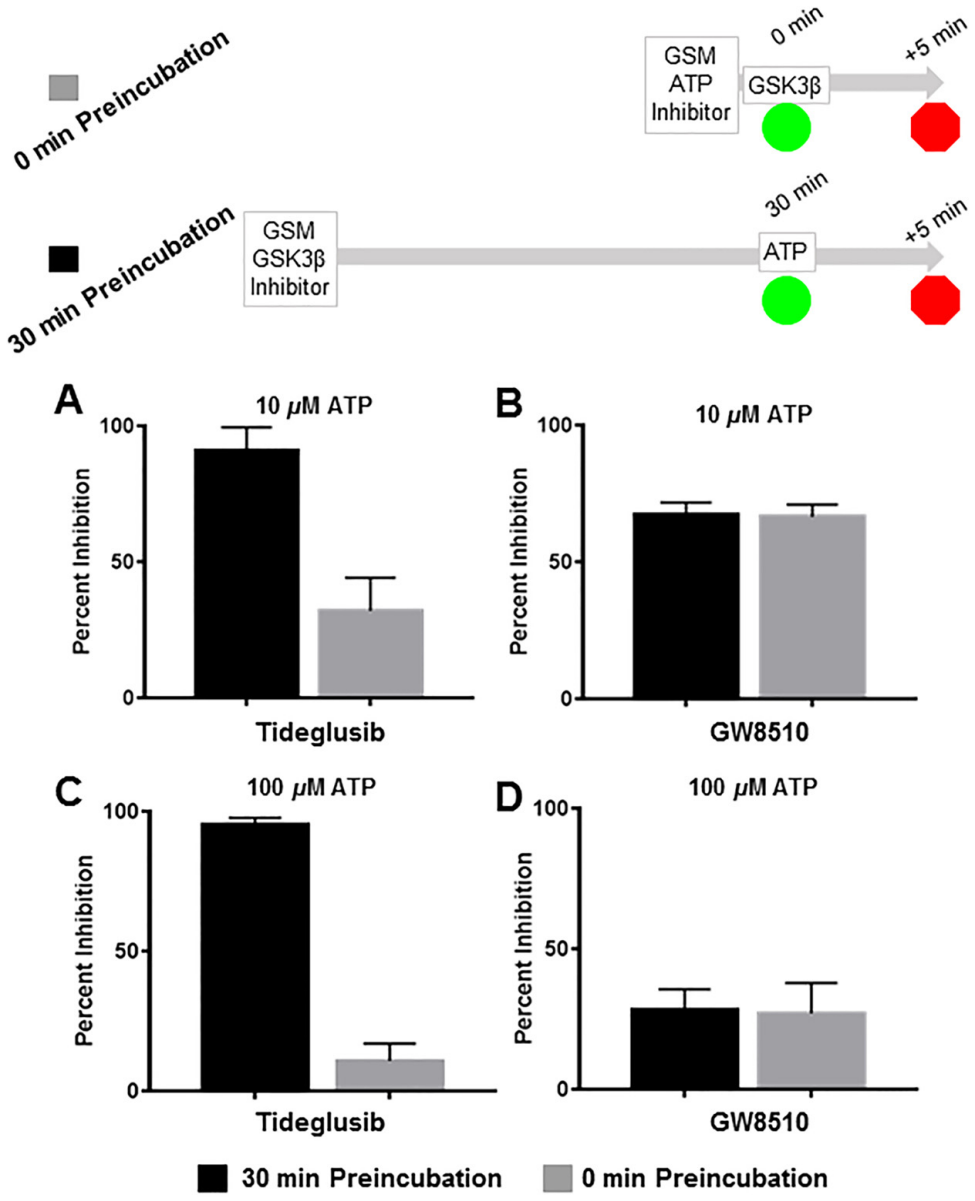
Four point MMOA screen for tideglusib and GW8510. Time dependent inhibition was evaluated by preincubation of TbGSK3β with 60 nM tideglusib and 6 nM GW-8510 with 10μM and 100μM ATP. **(A)**. Tideglusib [60 nM] in 10μM ATP. **(B).** GW8510 [60 nM] in 10μM ATP. (C.) Tideglusib [60 nM] at 100μM ATP. **(D.)** GW8510 [60 nM] at 100μM ATP. All reactions preincubated or not preincubated with TbGSK3β for 30 min at room temperature. Experiments run with 10μM GSM peptide, 10μM ATP, and buffer. Minute preincubation (30 min) was preincubated with inhibitor, TbGSK3β, GSM peptide, and buffer. ATP was mixed to initiate reaction. No preincubation contained inhibitor, GSM peptide, ATP, and buffer. The reaction was initiated with TbGSK3β. Reactions were run at room temperature for 5 min and stopped at 80°C. ADP formed was measured by ADP-Glo kit. Values are mean +/- standard error. N = 3 for each experiment and experiments were run in duplicates. Control reactions contained DMSO and background was determined using a zero time incubation and subtracted from all reactions. Black = 30 min preincubation Grey = No preincubation.

The four point methodology was demonstrated for tideglusib and GW8510 (Fig 4). Tideglusib decreased the enzyme activity following preincubation (gray bars no preincubation; black bars 30 min preincubation) at both 10 μM and 100 μM ATP (Fig 4A and 4C, respectively). On the other hand there was no effect of GW8510 preincubation on the TbGSK3β activity at 10 μM and 100 μM ATP (Fig 4B and 4D, respectively). These results can be interpreted that GW-8510 binds to TbGSK3β corresponding to a one-step model, to from an EI complex (Fig 1) while tideglusib follows a two-step model, forming an initial EI complex that rearranges with time to a more stable E’I complex (Fig 1).

In ATP competition studies the percent inhibition of activity by GW8510 was less at higher ATP concentrations (100 μM) than lower ATP (10 μM), 28% vs 67%, respectively irrespective of preincubation time (Fig 4B and 4D). Tideglusib’s activity was also decreased at when there was no preincubation but not when there was a preincubation (100 μM) (Fig 4A and 4C). This suggested that 1) tideglusib’s initial binding to form EI was competitive with ATP, and 2) that there is a time-dependent transition from the EI state to a more stable E’I state and is not sensitive to ATP competition. Due to this transition, tideglusib is more potent than GW8510 at high ATP concentrations following preincubations. We did not observe a shift in tideglusib activity at higher concentrations [80 μM] of the peptide substrate, GSM (supplementary, S1 Fig) indicating that the tideglusib does not interact with the peptide substrate binding site.

### Progress curves

**Fig 5:**
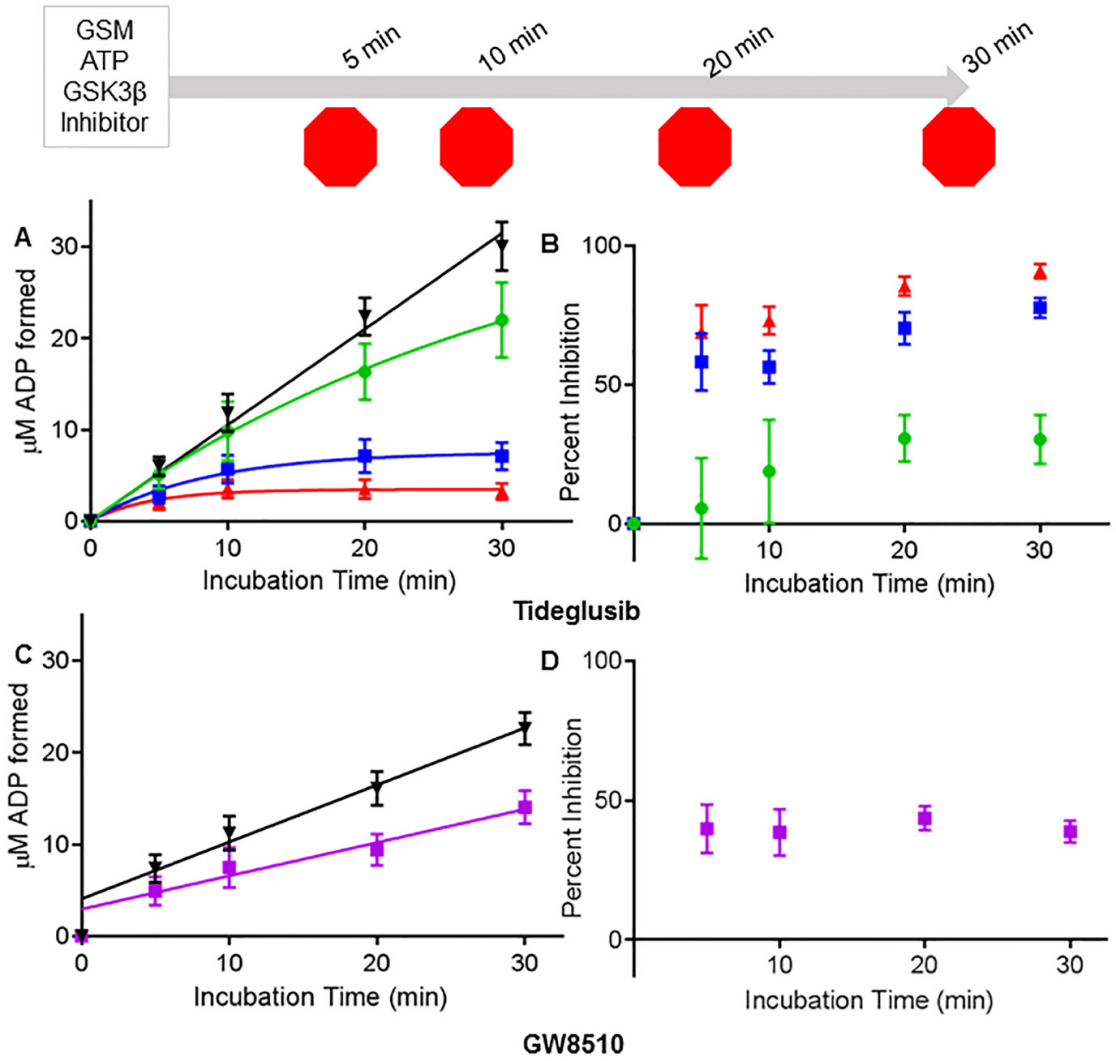
Progress curves for tideglusib and GW8510. Time dependent inhibition of TbGSK3β by tideglusib and GW8510 at 100 μM ATP and 80 μM GSM. A/B: TbGSK3β incubated with 0.2 μM (green circles), 0.4 μM (blue squares) or 0.6 μM (red triangles) tideglusib at 5, 10, 20, and 30 minute time intervals. C/D: TbGSK3β incubated with 0.6 μM (purple squares) GW8510 at 5, 10, 20, and 30 minute time intervals. All reactions were made up of 100 μM ATP, 80 μM GSM and buffer, mixed with inhibitor or DMSO. TbGSK3β [5 nM] was added to start reaction and incubated at room temperature at different time intervals. Reactions were stopped at 80°C and worked up as described in Materials and Methods. Data in A best fit the progress curve for irreversible inhibition (P = v_s_t + (v_0_ –v_s_) (1-e^-kt^)/k) where v_s_ equals zero. In C the data fit a straight line as expected for reversible inhibition, however the y-intercept was above zero. Values are mean +/- SE. All experiments run in duplicates. A. ADP formed with time (N = 6). B. Replot of data in A expressed as percent inhibition by tideglusib with time. C. ADP formed with time (N = 6). D. Replot of data in A expressed as percent inhibition by GW8510 with time.

Further evaluation of time dependence under more physiological conditions of no preincubation and higher ATP concentration was evaluated using enzyme progress curves. Progress curves measure the progress of the enzyme reaction with time [28]. In these reactions the time-dependent inhibition of TbGSK3β by tideglusib and GW8510 was evaluated under conditions in which inhibitor, 100 μM ATP and 80 μM GSM were combined in buffer and the reactions started with TbGSK3β (Fig 5). The reactions were stopped by heating at the specific times (5, 10, 20, 30 and 60 min). The progress of the reaction, as measured by product formation (ADP) with no inhibitor (DMSO control), was linear with time. When tideglusib was added, the percent inhibition was greater at 30 min as compared to 5 min, consistent with time-dependent loss of activity. In contrast, the effect of GW8510 on the rate of product formation was similar at all time points, consistent with rapid equilibrium reversible inhibition. Also of interest with tideglusib is that the slope of the progress curves approached zero at the later incubation times in a concentration dependent manner, suggesting complete inhibition of the enzyme at the later time points at all concentrations, behavior consistent with irreversible inhibition (Fig 5A).

### Irreversible behavior of tideglusib

**Fig 6:**
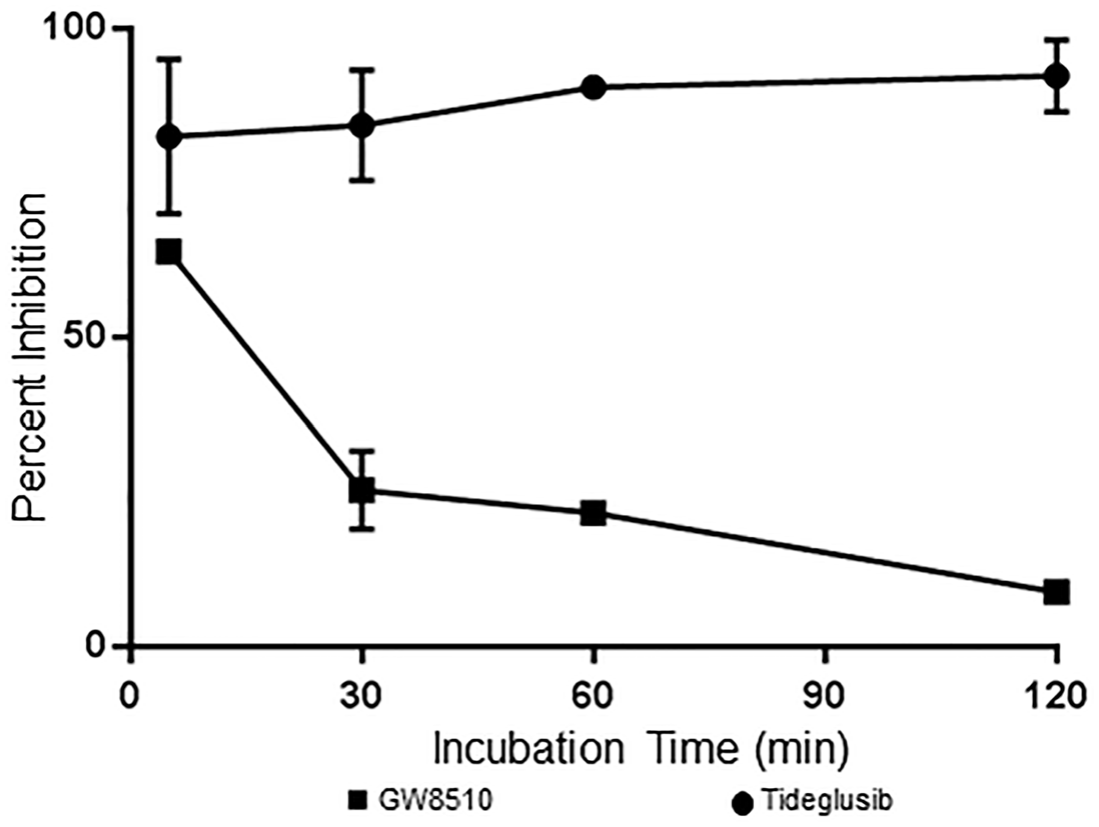
Tideglusib retains activity following a 100-fold dilution of TbGSK3β. Tideglusib [100 nM, GW8510 [100 nM] and DMSO were preincubated with 50 nM TbGSK3 for 30 min. After a 100 fold dilution activity was measured and compared to DMSO containing reaction. Data expressed percent inhibition of DMSO activity. Data represent the average of two separate experiments for GW8510 and three for tideglusib. All points in each experiment were run in duplicate.

Tideglusib and GW8510 were evaluated for irreversibility by measuring the TbGSK3β activity following a 100-fold dilution with reaction buffer. In these experiments tideglusib and GW8510 [100 nM] were preincubated with 50 nM TbGSK3β for 30 min at which time the reaction was diluted 100 fold into a solution containing 100 μM ATP and 80 μM GSM. The activity was then measured for up to two hours (Fig 6). The activity was compared to a DMSO control (also preincubated for 30 min and activity measure up to 2 hr). The final concentration of tideglusib and GW8510 following dilution was 1 nM. Inhibition of enzyme activity was retained following dilution of tideglusib whereas enzyme activity was recovered in the reactions with GW8510. The results are consistent with reversible behavior of GW8510 and irreversible behavior of tideglusib. This experiment does not distinguish between a very slow dissociation rate or covalent binding. As discussed below, tideglusib was previously demonstrated to have irreversible inhibition against human GSK3β with an IC_50_ of 5 nM following preincubation, with no evidence for covalent binding [29]. These investigators concluded in that work that tideglusib was a functionally irreversible inhibitor [29]. The results shown in Fig 6 are consistent with similar behavior for TbGSK3β.

### Mechanistic screening of TbGSK3β inhibitors

**Fig 7:**
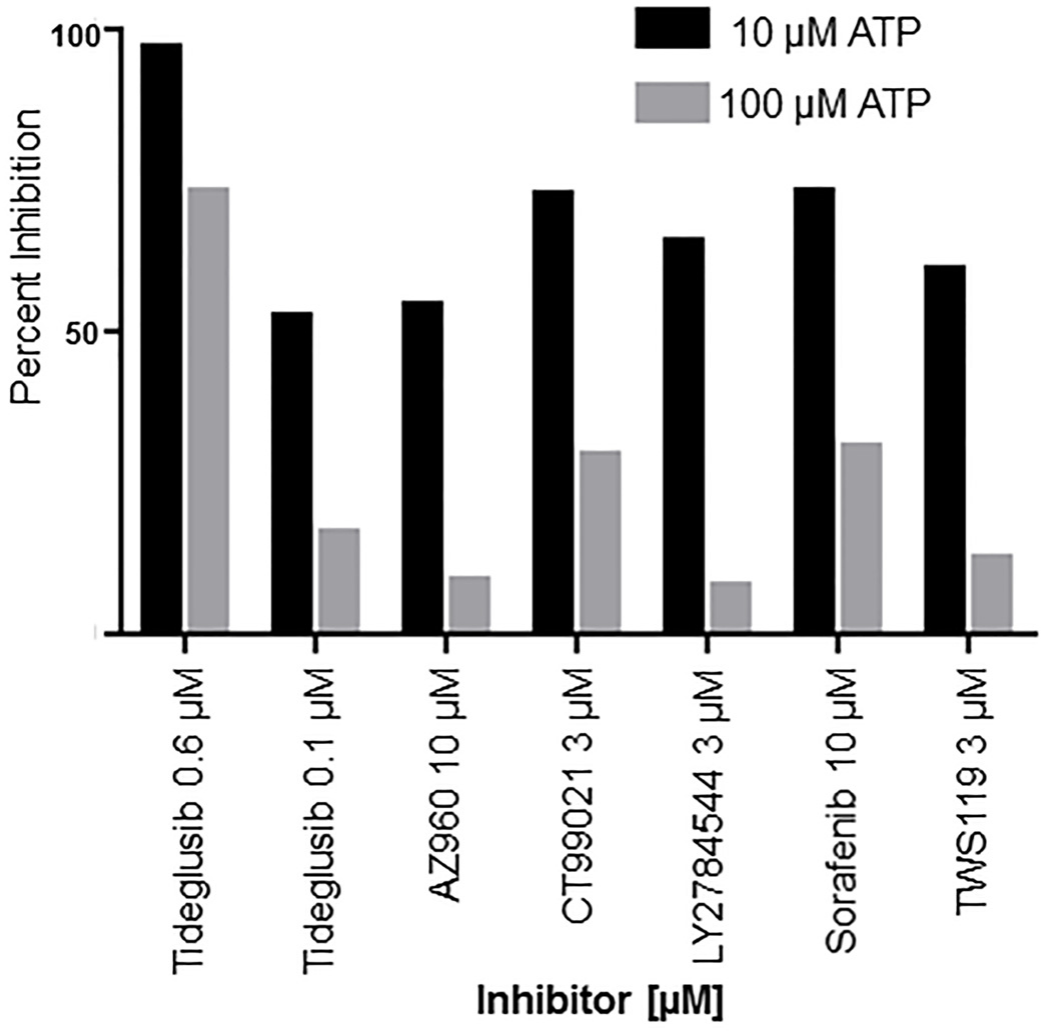
Two point ATP competition analysis for TbGSK3β inhibitors. Tideglusib, AZ960, CT99021, LY2784544, sorafenib, and TWS119 at 10μM GSM and 10 μM or 100 μM ATP. GSM mixed with 1x buffer and 10μM or 100μM ATP. TbGSK3β [5 nM] was added to start reaction and incubated at RT for 5 minutes. Reactions were stopped at 80°C and worked up as described in Materials and Methods. Experiments ran in duplicate, N = 2.

The four point MMOA assay was used to characterize molecules that had previously been identified as TbGSK3β inhibitors. The concentrations chosen for the MMOA assays were determined from the IC_50_ in the HTS screens. A good starting concentration for the time-dependent studies is the IC_25_ (concentration at 25% inhibition), since time-dependent inhibition will increase with preincubation time. For the competition analysis, use of an inhibitor concentration equal to the IC_50_ at substrate Km (theoretically 2x Ki) will allow observation of the decrease in inhibition due to higher substrate concentrations (Fig 5). In practice, concentrations must be chosen that will allow the change to be observed and will be specific to each inhibitor. The activities of these molecules were not time dependent since they were unchanged after 0 and 30 minutes incubation (Table 1) and all were found to be competitive with ATP since the percent inhibition was less using 100 μM versus 10 μM (Fig 7).

**Table 1:** Evaluation of time dependent inhibition withTbGSK3β inhibitors. Tideglusib, AZ960, CT99021, LY278544, sorafenib, and TWS119 were evaluated for time-dependent activity. The procedures are described in Fig 4. Data are the mean of duplicate experiments with N = 2 for each experiment. Data shown are percent inhibition of DMSO control. Ratio is % inhibition at 30 min preincubation/ % inhibition at 0 min preincubation at 10 μM ATP. Concentrations were selected based on preliminary screening IC_50_ measurements with no preincubation. IC_50_ values ± standard deviation +n = 3) were determined with 10 μM ATP and 10 μM GSM.

Compound	IC_50_	conc^a^	0 min preincubation	30 min preincubation	Ratio Preinc/no preinc
	μM	μM	% inhibition	% inhibition	
Tideglusib	0.17± 0.023	0.06	6.2	98	16
AZ960	4.1±1.27	10	55	40	0.73
CT99021	0.2±0.08	3.0	74	72	0.97
LY278544	0.5±0.12	1.0	36	30	0.83
Sorafenib	1.7±0.21	3.0	18	26	1.4
TWS119	0.6±0.14	1.0	35	35	1

^a^Concentration of inhibitors tested with and without preincubation to determine the ratio.

### Activity in parasites

**Fig 8:**
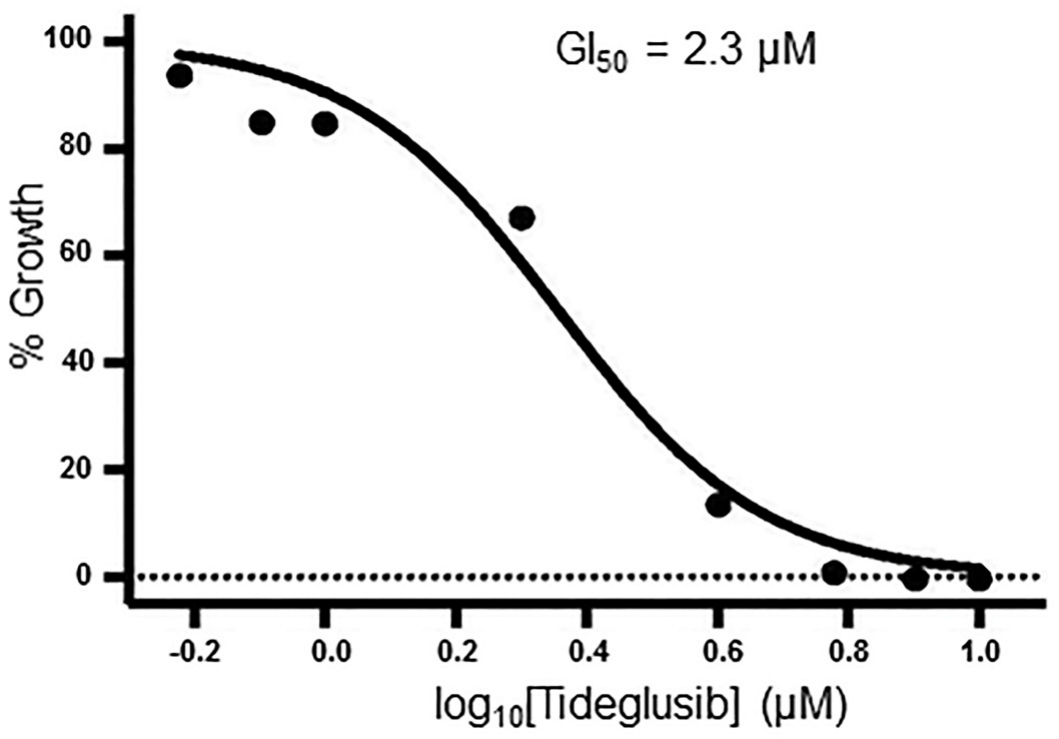
Tideglusib inhibits growth of *T*. *brucei*. Bloodstream trypanosomes (3 x 10^3^ cells/mL) were treated for 48 hours at varying concentrations of tideglusib (in DMSO solvent, final concentration of DMSO was 0.1%). Percent growth was determined by comparison to trypanosomes treated with equivalent volume of solvent (0.1% DMSO). Non-linear regression fit of the dose-response curve was determined using GraphPad Prism 6 software.

Tideglusib was evaluated in *T*. *brucei* parasite assays as previously described in Materials and Methods. The concentration of inhibitor at which trypanosome proliferation was inhibited by 50% (GI_50_) was 2.3 μM (Fig 8). In comparison, Ojo and coworkers reported the activity of GW-8510 in a similar 48 hr parasite growth assay with *T*. *brucei brucei* strain 427 to have an EC_50_ of 119 nM [30].

**Fig 9:**
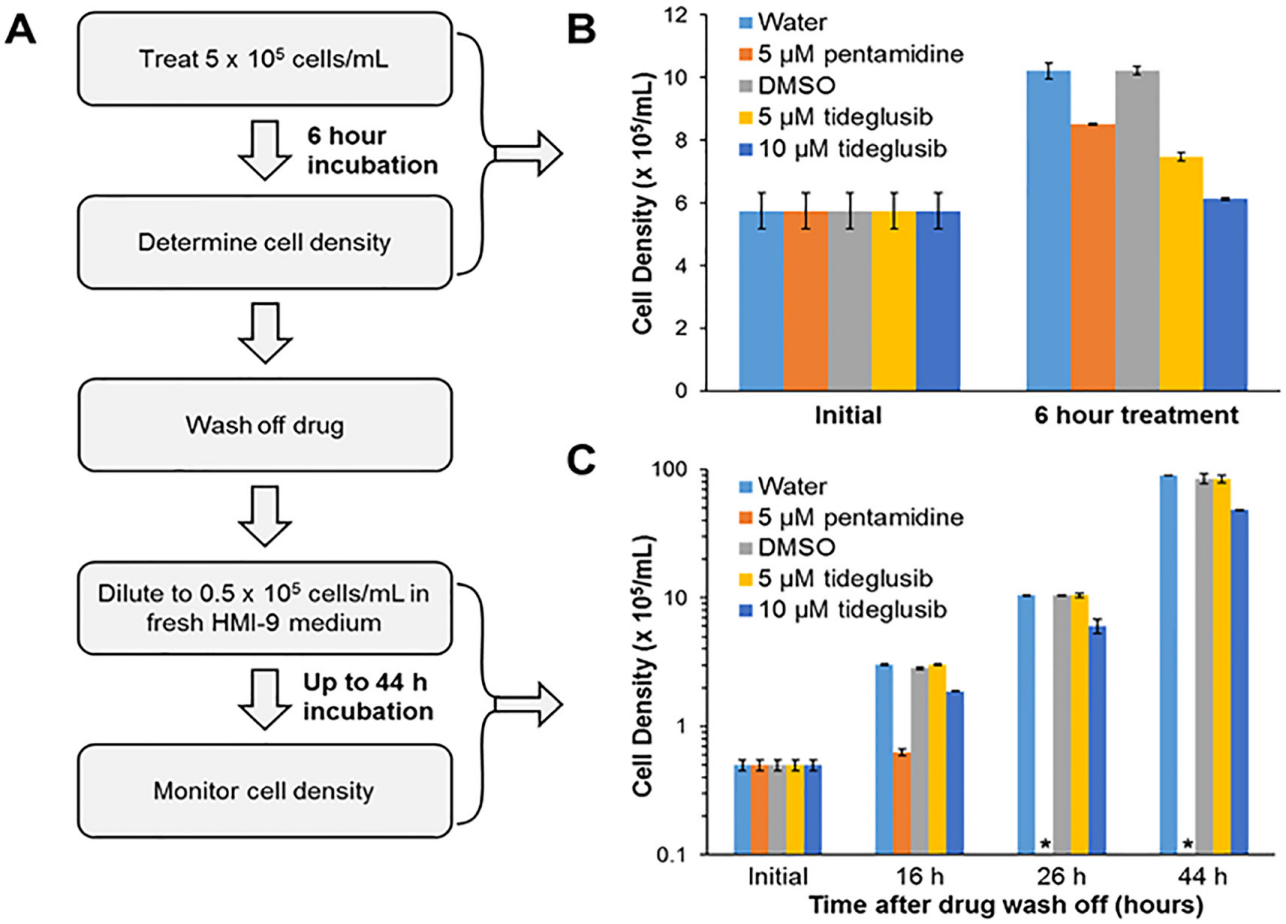
Trypanosome viability after short term exposure with tideglusib. Trypanosomes (5 x 10^5^/mL in HMI-9 medium) were treated with pentamidine (or water solvent control) or tideglusib (5 μM, 10 μM, or DMSO solvent control) for 6 hours (37°C, 5% CO_2_) and then cultured in drug-free treatment as detailed in panel **(A)**. Trypanosome densities were determined after 6 hour treatment **(B)**, and the drugs washed from the cells by centrifugation and resuspension in fresh medium (to a density of 0.5 x 10^5^/mL). Trypanosome proliferation in the fresh medium was monitored for 44 hours (37°C, 5% CO_2_) **(C)**. Error between four separate cultures are shown. Cultures in which no cells were observed is denoted by an asterisk.

The effect of tideglusib on trypanosome viability after short term exposure was evaluated to determine if the molecule is cidal or cytostatic. During the short 6 hour treatment at high trypanosome density (5 x 10^5^/mL), tideglusib (at 5 or 10 μM) markedly slowed trypanosome growth (Fig 9B). In fact at 10 μM tideglusib, proliferation was entirely halted. However, dilution of tideglusib (or DMSO) from the media no longer arrested trypanosome proliferation, with a slight lag in recovery after 10 μM tideglusib exposure. After the initial lag in recovered growth, the cells grew with a normal rate of approximately 11 doublings in 48 hours (Fig 9C). This is in contrast to the control, pentamidine, which killed all observed trypanosomes within 24 hours of the 6 hour exposure (Fig 9C).

These results show that tideglusib was cytostatic at high trypanosome cell densities. Trypanosomes treated with 10 μM tideglusib did demonstrate a slight lag in normal proliferation rates after the drug pressure was removed (Fig 9C). We conclude that tideglusib is better at controlling proliferation during short exposure, but pentamidine wins over the long run.

## Discussion

As part of our on-going efforts to identify new treatment molecules for NTDs we have been investigating approaches to identify new MMOAs early in the discovery process. Currently the identification of MMOA occurs after a molecule has been identified as a lead or clinical candidate using rigorous biochemical and pharmacological methods. Previous analyses have suggested that an optimal MMOA can provide an improved therapeutic index, however the challenge is to identify compounds with an optimal MMOA [6, 31]. One approach to accomplish early identification of MMOA is to identify clusters of compounds with different MMOAs. MMOA identification would be used in addition to other factors to cluster molecules, such as activity, chemical structure and physical properties. Representatives from these compound clusters will then be evaluated empirically in phenotypic screens to identify actives and new opportunities. Accordingly, simple, efficient methods are required to easily identify molecules with different mechanistic features. We report here a simple, efficient four point MMOA screening method for evaluating compounds for time-dependent activity and competitive inhibition.

In practice, this four point MMOA method can be applied at any stage in the drug discovery process. In early discovery, hits are identified in screens at one or a few concentrations and confirmed with IC_50_ determinations. The IC_50_ concentration will inform the concentration to use for the four point MMOA method. It should be noted that this method can be employed with any enzyme, regardless of the number of substrates by using two concentrations of the substrate of interest (one around Km and the other above Km).

It is worth mentioning that this four point MMOA method is a simplification of more rigorous methods that are common practice in biochemistry and pharmacology labs. Typically studies to look for time-dependence and competition are accomplished by investigating complete dose-response curves whereby the results inform a detailed understanding of the molecular interaction. In preparation for these definitive studies, smaller experiments are used to establish conditions. We have found that the four point MMOA studies are suitable for screening purposes and can be sufficiently reliable to differentiate compounds. In previous work, we applied a similar strategy to differentiate kinetics for antagonists binding to CCR5 using IC_50_ measures in receptor competition assays with and without preincubation [32]. Guo *et al* have recently described a dual point competition association assay for assessing ligand-receptor binding kinetics. Their approach was found to be useful for ranking molecules [33]. The four point MMOA method described here is a simpler method as compared to both of these, requiring less data points, thereby resulting in more efficient use of time and resources. Interesting findings from the four point MMOA method can be evaluated in more detailed assays as warranted.

Using the four point MMOA method, tideglusib was determined to be a time-dependent inhibitor of TbGSK3β. Its effectiveness increased with time until its inhibition activity was similar to GW8510, a well-documented potent TbGSK3β inhibitor [30](Figs 3 and 4). Both tideglusib and GW8510 competed with ATP for binding to TbGSK3β without preincubation. Interestingly, tideglusib’s inhibition increased with preincubation time (Figs 3 and 4), incubation time (Fig 5) and showed non-competitive behavior following preincubation (Fig 4). In comparison, the MMOA of GW8510 was competitive following preincubation (Fig 4). These results show tideglusib to have a different mechanistic profile for TbGSK3β than GW8510. Tideglusib shows a time dependent switch from competitive to non-competitive behavior. This type of behavior is seen with other slow-dissociating molecules, which has been termed insurmountable and is a property of successful medicines [5, 17, 19, 20, 34]. We also used the four point MMOA method to characterize other kinase inhibitors that were previously identified in screens as TbGSK3β inhibitors (Fig 7, Table 1). All were found to behave mechanistically as non-time dependent, competitive inhibitors.

Adjusting the enzyme assays to account for these mechanisms showed that GW8510 and tideglusib had similar activity for TbGSK3β under more physiological conditions of preincubation and 100 μM ATP (Fig 4). However, this similarity did not translate to cellular activity, where GW-8510 was more active than tideglusib (0.12 μM to 2.3 μM, respectively [29]). These data suggests that factors other than the TbGSK3β MMOA differentiate the effect of these molecules against *T*. *brucei*, and demonstrates the value of early MMOA determination to help understand target specific hypotheses. Many factors may contribute to the difference in activity including poor solubility of tideglusib that limits cellular activity and/or that the cellular activity of GW-8510 maybe due in part to a non-TbGSK3β mechanism. While further studies are needed to evaluate these issues, the understanding of their different TbGSK3β MMOAs will assist in designing and correctly interpreting the data.

The MMOA for tideglusib inhibition of TbGSK3β identified with the four point MMOA assay is consistent with that observed for inhibition of human GSK3β as reported by Dominquez and coworkers [29]. Using a more sophisticated analysis they also reported a binding mechanism which had an ATP-competitive component [29]. Furthermore, their studies on time-dependence led to the conclusion that tideglusib is a functionally irreversible inhibitor of human GSK3β and that the duration of the pharmacological effect caused by this behavior may be exploited to maximize its therapeutic potential. The work described here for TbGSK3β inhibition by tideglusib shows that the mechanism of inhibition is similar against the enzymes from human and *T*. *brucei*.

In humans, GSK3β is a regulatory kinase for over 40 different proteins in a variety of pathways, and has been implicated in a number of diseases including: Type II diabetes (Diabetes mellitus type 2), Alzheimer's Disease, inflammation, cancer, and bipolar disorder [26]. Furthermore, studies have shown that one of the *T*. *brucei* GSK3 homologs (*Tb*GSK3 short), is necessary for cell growth and viability and thus may serve as a potential drug target for the treatment of HAT [30]. In a recent clinical trial designed to investigate safety and efficacy, tideglusib administered in escalating doses of up to 1000 mg/day to 30 Alzheimer’s patients for 6 weeks was generally well tolerated [25]. Tideglusib was also well-tolerated in phase II studies of Alzheimer’s disease and progressive supranuclear palsy [35, 36]. Blood levels for tideglusib were reported in mice of approximately 2.25±1.55 μg/ml (6.7 μM) after oral administration [37].

In summary, we used the four point MMOA method to evaluate the MMOA for *T*.*brucei* GSK3β inhibitors and observed tideglusib to have a time-dependent mechanism which differentiated it from rapidly reversible inhibitors, such as GW8510. Tideglusib was shown to inhibit parasite growth in this work and has been reported to be well tolerated in one year of dosing in human clinical studies [35–37]. Consequently, further supportive studies on the potential therapeutic usefulness of tideglusib for HAT are justified.

## Supporting Information

S1 Fig Four point MMOA screen for tideglusib with respect to peptide substrate. Time dependent inhibition by preincubation of TbGSK3β with tideglusib in 10 μM and 80 μM GSM peptide. **(A)**. Tideglusib in 10 μM GSM. **(B).** Tideglusib in 80 μM GSM. All reactions preincubated or not preincubated with TbGSK3β for 30 min at room temperature at 0.06 μM. Experiments run with 10 μM ATP. Assays with 30 min preincubation were preincubated with inhibitor, TbGSK3β, GSM peptide, and buffer. ATP was mixed to initiate reaction. 0 minute preincubation contained inhibitor, GSM peptide, ATP, and buffer. TbGSK3β was mixed to initiate reaction. Reactions were run at room temperature for 5 minutes and stopped at 80°C. ADP formed was measured by Promega ADP-Glo kit. Black = 30 minute preincubation Grey = 0 minute preincubation
(TIF)
