# Supplementary figures and images for: A Four-Point Screening Method for Assessing Molecular Mechanism of Action (MMOA) Identifies Tideglusib as a Time-Dependent Inhibitor of Trypanosoma brucei GSK3β
Source: PLoS Negl Trop Dis. 2016 Mar 4;10(3):e0004506. doi: 10.1371/journal.pntd.0004506 (PMC4778863; doi:10.1371/journal.pntd.0004506)

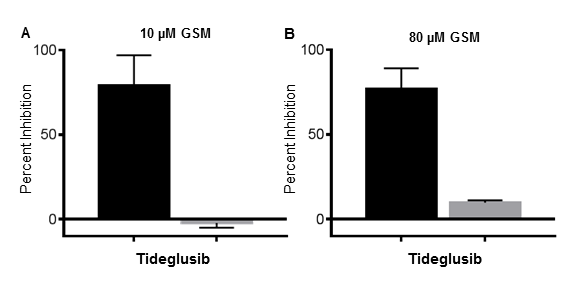

Supplement: S1 Fig — Time dependent inhibition by preincubation of TbGSK3β with tideglusib in 10 μM and 80 μM GSM peptide. (A). Tideglusib in 10 μM GSM. (B). Tideglusib in 80 μM GSM. All reactions preincubated or not preincubated with TbGSK3β for 30 min at room temperature at 0.06 μM. Experiments run with 10 μM ATP. Assays with 30 min preincubation were preincubated with inhibitor, TbGSK3β, GSM peptide, and buffer. ATP was mixed to initiate reaction. 0 minute preincubation contained inhibitor, GSM peptide, ATP, and buffer. TbGSK3β was mixed to initiate reaction. Reactions were run at room temperature for 5 minutes and stopped at 80°C. ADP formed was measured by Promega ADP-Glo kit. Black = 30 minute preincubation Grey = 0 minute preincubation (TIF) [file pntd.0004506.s001.tif]
